# Monitoring and Scoring Counter-Diffusion Protein Crystallization Experiments in Capillaries by *in situ* Dynamic Light Scattering

**DOI:** 10.1371/journal.pone.0033545

**Published:** 2012-06-04

**Authors:** Dominik Oberthuer, Emilio Melero-García, Karsten Dierks, Arne Meyer, Christian Betzel, Alfonso Garcia-Caballero, Jose A. Gavira

**Affiliations:** 1 Institute for Biochemistry and Molecular Biology, Laboratory for Structural Biology of infection and inflammation, University of Hamburg, Hamburg, Germany; 2 Laboratorio de Estudios Cristalográficos, IACT (Consejo Superior de Investigaciones Científicas-Universidad de Granada), Granada, Spain; 3 Institute of Biochemistry, Centre for Structural and Cell Biology in Medicine, University of Lübeck, Lübeck, Germany; Russian Academy of Sciences, Institute for Biological Instrumentation, Russian Federation

## Abstract

In this paper, we demonstrate the feasibility of using *in situ* Dynamic Light Scattering (DLS) to monitor counter-diffusion crystallization experiments in capillaries. Firstly, we have validated the quality of the DLS signal in thin capillaries, which is comparable to that obtained in standard quartz cuvettes. Then, we have carried out DLS measurements of a counter-diffusion crystallization experiment of glucose isomerase in capillaries of different diameters (0.1, 0.2 and 0.3 mm) in order to follow the temporal evolution of protein supersaturation. Finally, we have compared DLS data with optical recordings of the progression of the crystallization front and with a simulation model of counter-diffusion in 1D.

## Introduction

The crystallization of any given protein [1], [2], [3] is a process that can be outlined in three successive and interrelated stages. Firstly, a purified solution of the target protein must be brought into a supersaturated state [4], which is characterized by a ratio of concentration-to-solubility higher than one. The rate at which supersaturation is achieved is one of the most important parameters in protein crystallization since it defines a very specific temporal pathway through the phase diagram [5]. The supersaturation state can be achieved either immediately using the batch technique [6], progressively by means of vapour-diffusion methods [7], [8] or continuously as with the counter-diffusion technique [9]. Secondly, at least one or more nucleation events will take place. The nucleation step [5] can be understood as a process by which, stochastically in space and time, a new phase with lower chemical potential appears in solution. In other words, stable protein nuclei are formed from a dynamical size distribution of density fluctuations that are driven by the supersaturated state of the solution. In the last step, crystal growth, the stable aggregates or nuclei that have formed during nucleation start to grow at the expense of the remaining protein in solution. The growth of crystals consumes the protein, thereby reducing the supersaturation value of the solution and driving the system back towards equilibrium.

Control of the nucleation stage of a crystallization experiment is crucial in order to obtain large quality crystals, representing a more rational approach than the use of automated large-scale high throughput screening strategies. Indeed, the dynamics of nucleation and cluster formation, which can be studied by several techniques such as SAXS, SANS [10] or Dynamic Light Scattering (DLS) [11], are currently a subject of much debate [12], [13]. In recent years, light scattering techniques [14], [15] have emerged as one of the most promising techniques to investigate and monitor the behaviour of nucleating protein solutions. In particular, DLS [16], [17] has been used to obtain information about the detailed physico-chemical processes that occur during nucleation and early stages of crystallization [18], [19], [20], [21] or to screen for crystallization conditions [22], [23] and crystallizability of biological macromolecules [24], [25]. While static light scattering (SLS) provides detailed information on parameters such as the average molecular weight and the second virial coefficient [17], [26], DLS yields data on the size distribution of protein aggregates and can thus be used to follow protein aggregation in crystallizing solutions [14], [15]. Recently, it has been shown that DLS can also be employed to determine interaction parameters [27], [28] leading to similar information as obtained by the second virial coefficient. Hence the application of light scattering methods in standard crystallization experiments (such as vapour diffusion [14], [29], [30], [31] or as described here: counter-diffusion) is a step forward towards controlled and efficient crystallization of biological macromolecules.

The method of Counter-diffusion (CD) applied here has a long history [32]. Already in 1972 F.R. Salemme showed that proteins could be crystallized by free-interface diffusion within glass capillaries [33]. CD is an excellent method to optimize crystallization trials and to obtain high quality crystals because a large number of conditions (ratio of precipitant/protein concentrations) are continuously self-screened per capillary experiment [9]. Moreover, the use of small capillaries (0.1 mm inner diameter) in counter-diffusion trials is a very attractive experimental option since it saves protein sample; each capillary experiment only requiring around 400 nanolitres of protein solution. Nonetheless, the complex spatio-temporal evolution of supersaturation that takes place during a counter-diffusion experiment [34] makes it difficult to predict what is happening and to exert some degree of control over it, which is not necessarily required for screening purposes but desired for the purpose of improving crystal quality. In this context, DLS would enable to follow the evolution of counter-diffusion experiments *in situ* in thin capillaries non-invasively. In fact, DLS has already been used in combination with other crystallization techniques such as vapour diffusion and batch in commercially available crystallization plates owing to the introduction of the *Spectro*LIGHT 500 instrument developed by Nabitec GmbH [14]. In contrast, the application of DLS to analyse capillary counter-diffusion experiments is more complex since the volume in which to measure is relatively small (capillaries of 0.1 mm diameter) and the optical properties of the thin glass are more complex than those of quartz cuvettes or droplets.

The aim of this work is to demonstrate the feasibility of applying DLS to monitor *in situ* the evolution of counter-diffusion experiments in thin capillaries down to 0.1 mm of inner diameter GCB-D [35]. We have selected Glucose isomerase from Streptomyces rubiginosus (EC 5.3.1.5.) for the crystallization experiments because it is a well characterized, very stable enzyme [36] that has been previously used in crystallization studies [37], [38], [39] and as a standard marker in SAXS experiments [40], among other studies. DLS data have been systematically obtained, analysed and compared with video microscopy recordings of the evolution of crystallization experiments along the capillaries. The experimental results have also been compared with theoretical data calculated by a programme simulating the diffusion-precipitation coupling effect that controls the evolution of supersaturation inside the capillary.

## Results and Discussion

### *In situ* DLS Measurements in Capillaries

**Figure 1:**
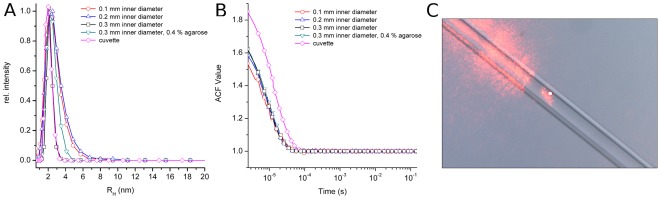
Comparison of DLS measurements in thin capillaries and a quartz cuvette. A) It shows an overlay plot of the radius distribution of glucose isomerase in capillaries and a quartz cuvette; B) It shows an overlay of the auto correlation function from which the radius distribution were derived; C) Picture showing the focus of the laser inside a 0.1 mm capillary and the scattering associated to the capillary-walls, which does not affect the measurements.

The volume of solution analysed by the DLS system (crossing space of laser and detector) has been previously estimated at around 1 pL [14]. Such volume is equivalent to a sphere of approximately 12.5 micrometres in diameter and should not be neglected for measurements in 100 micrometres capillaries that, on the other hand, are the most widely used because of its small sample consumption (approximately 400 nL). Therefore, initially it was necessary to establish whether it is possible to obtain a clean DLS signal similar to that obtained in quartz cuvettes without any special distortion due to the geometry of the capillary or the plastic walls of the GCB-D. Different sets of DLS data of a protein solution were taken in a quartz cuvette and capillaries of varying diameters for comparative purposes. Three capillaries of 0.1, 0.2, of 0.3 mm plus an extra capillary of 0.3 mm with agarose were filled with a solution of glucose isomerase in its buffer and subsequently punctured in GCB-D’s filled with 3 M ammonium sulphate. A DLS measurement was taken at 30 mm from the open end of the capillaries to make sure that the precipitant had not reached that position. Then, a total of 15 DLS measurements at 2 seconds interval were taken for each capillary and the quartz cuvette. The results of the size distributions obtained from the DLS signal are shown in Figure 1.

Figure 1C shows how the focus of the DLS laser beam fits inside the capillary, being sufficiently away from the flares caused by the reflection of the laser on the walls of the GCB-D, even for the smallest 0.1 mm capillaries. In Figure 1A, all the series of measurements exhibit monomodal size distribution, that is, there is only one clear peak that corresponds to the hydrodynamic radius of glucose isomerase in the buffer without precipitant. Figure 1B shows the auto correlation functions (ACF) [17] of DLS measurements within capillaries of 300, 200 and 100 µm inner diameter in comparison with an ACF derived by DLS measurements in a quartz cuvette; although the signal-to-noise ratio is slightly better in the cuvette than in capillaries, all ACFs are comparable and show that valid DLS measurements can be taken from thin capillaries. In summary, the results shown in Figure 1 validate the quality of DLS measurements in standard commercial counter-diffusion devices even in the presence of low agarose concentration.

### DLS Monitoring of a Counter-Diffusion Crystallization Experiment

**Figure 2:**
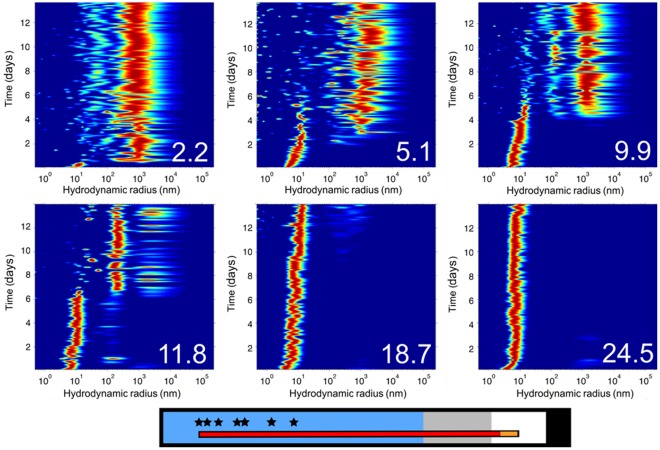
Pictures of the size distribution (X-axis) as a function of time (Y-axis) obtained from DLS measurements in a single capillary. The number on the bottom right corner of each picture denotes the distance of the measurement in millimetres from the open end of the capillary. Time is displayed in arbitrary units (a.u.), where each unit corresponds to 1/10 of a day. The picture at the bottom shows an overview of the capillary and the position of the measurements (*) in the GCB-D.

After validating the use of DLS in capillaries, we set up a crystallization experiment of glucose isomerase in a capillary of 0.1 mm and followed the evolution of size distribution of the protein as a function of time and distance inside the capillary (see Figure 2).

The start of the counter-diffusion experiment was set (time = 0) when the open-end of the capillary is punctured in the precipitant solution, moment at which the protein and precipitant start to diffuse against each other. The precipitant moves faster than the protein due to its smaller diffusion coefficient, approximately one order of magnitude lower. As the precipitant diffuses along the capillary, the solubility of the protein decreases and precipitation takes place (see Movies S1 and S2).

Two distinct effects were observed in the calculated size distribution of glucose isomerase. Firstly, there is a slight shift of the hydrodynamic radius from 2 nm to approximately 5 nm within the first 2–3 days after the start of the experiment, which is associated to the formation of the glucose isomerase tetramer. Secondly, at a certain point in time–that is different for each measured position in the capillary–the peak of the protein in solution disappears giving rise to a new broader peak at larger diameters (100–1000 nm). The quick disappearance of the initial protein peak is an indication of crystallization events [14] taking place progressively later in time at larger distances from the entrance of the capillary. Such observation is in agreement with both the simulation results and the theoretical formation and evolution of the advancing supersaturation wave [41] produced by the continuous diffusion of the precipitant. Furthermore, the irregular size distributions at larger hydrodynamic radii are a result of the perturbation of the DLS signal due to the presence of large aggregates.

As the distance of measurement from the open end of the capillary gets bigger, the crystallization events are not so clearly detected by DLS. This is because the processes of nucleation and crystal growth at such positions occur at lower supersaturation values. Since nucleation and crystal growth are the cause of the depletion of the protein, we may conclude that at longer distances within the capillary these processes are not fast and/or intense enough to counteract the restoration of the consumed protein by diffusion from the rest of the capillary. Nonetheless, the increase of precipitant concentration due to diffusion is still observed from the shift of the protein peak.

**Figure 3:**
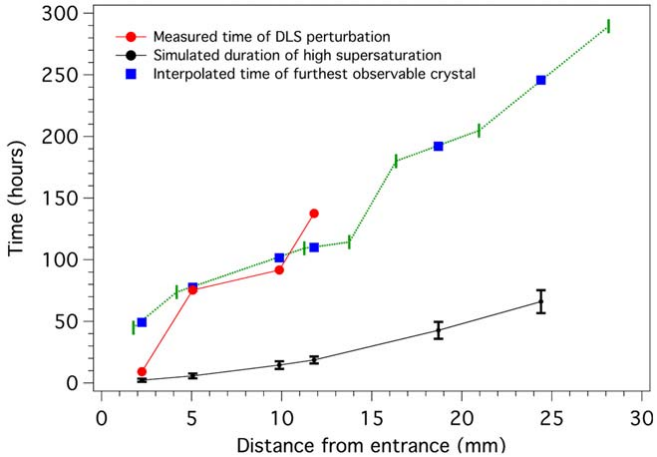
Comparison of data obtained from DLS measurements, optical microscopy records and the numerical simulation. The data at 18.7 and 24.5 mm have not been drawn because it does not show disturbance of the size distribution. The green horizontal line corresponds to original optical microscopy data plotted as ‘time *vs* distance’ instead of ‘distance *vs* time’, using the same limits as those used to plot the DLS and the simulation data. The axis of the original optical microscopy data have not been drawn for clarity.

In order to compare DLS data with optical microscopy observations, we prepared a counter-diffusion experiment under identical conditions and recorded the progress of the crystallization process by registering the distance of the furthest observable crystal from the entrance of the capillary at different times. Figure 3 illustrates how the disturbance observed in the size distribution at each position occurs earlier in time than the interpolated appearance of crystals, as expected. Only at the last point of measurement, where we know that the nucleation rate is already low, the perturbation of the DLS signal took a bit longer to be detected.

We have compared the experimental DLS data with our simulation software [42], [43] that calculates supersaturation values of the system in the capillary and its evolution as a function of distance and time. The numerical code of the software takes into account diffusion mass transport, nucleation and crystal growth in one dimension, and depends upon some geometrical, physical and chemical parameters, which have been described in the experimental section. In Figure 3 we have plotted the duration of conditions of relatively large supersaturation (σ>1.5) at the positions monitored by DLS. Since the probability of nucleation depends on a power of the natural logarithm of supersaturation, we would expect that nucleation would occur during the period in which the system is supersaturated, followed by crystal growth that could be detectable by DLS. However, our simulation calculates the time interval where the system is supersaturated earlier than it should according to DLS and optical observations. This discrepancy may be due to the algorithm used, which does not take into account the fact that the diffusion of protein molecules is affected by the concentration of the precipitant. As a result, the diffusion obtained by the simulation software proceeds faster than it should and the system becomes supersaturated earlier than the detection of crystal growth by DLS and optical microscopy in the physical experiment. This information is very valuable as it could be used to improve the simulation algorithm and make a more accurate prediction of crystallization events.

All in all, we have developed a novel methodology that combines *in situ* DLS with the capillary counter-diffusion technique. The experimental results presented in this paper validate the use of *in situ* DLS to monitor counter-diffusion experiments in capillaries as small as 0.1 mm of inner diameter. In fact, the quality of the DLS signal taken in capillaries is similar to that obtained using standard quartz cuvettes. We have successfully monitored a real counter-diffusion experiment in standard GCB-D systems by *in situ* DLS allowing us to detect perturbations associated to crystallization events slightly before the appearance of optically observable crystals, especially at capillary positions closer to the beginning of the experiment. Furthermore, the DLS signal is sensitive to supersaturation changes inside the capillary, through the increase of the apparent protein hydrodynamic radius, which reflects the salt-dependent protein-protein interactions [27], [28].

The advantages of counter-diffusion for the crystallization of proteins are well-known and multiple: i) Screening of crystallization conditions is more efficient because several precipitants can be tried in one single capillary in a wide range of supersaturation and supersaturation rates (in practice this means that it is possible to obtain sequentially amorphous precipitation, microcrystals and crystals of the highest quality in a single capillary); ii) Cryo-protectants, heavy atoms, ligands or any other additive can also be diffused within the capillary/crystal in a mass transport diffusive regime; iii) The crystals grown can be diffracted directly from the capillary without further manipulation. The combination of *in situ* DLS in capillary counter-diffusion experiments presented in this paper adds two very important benefits. Firstly, it can provide valuable information about the stability of the protein, which is particularly useful for structural biologists. Secondly, capillary counter-diffusion experiments can be optimised using DLS data since it is possible to detect the nature of protein aggregates and their propensity to trigger nucleation and, hence, to identify whether one particular crystallisation condition is likely to produce crystals (i.e. by increasing protein concentration) or not.

## Materials and Methods

### Set-up of Crystallization Experiments with Glucose Isomerase

Glucose isomerase (EC 5.3.1.5) from *Streptomyces rubiginosus* is a homotetramer in solution with a molecular weight of 173 kDa [44]. Glucose isomerase was supplied by Hampton Research as a crystal suspension in 6 mM Tris-HCl pH 7.0, 0.91 M (NH_4_)_2_SO_4_, 1 mM MgSO_4_. The commercial product was extensively dialyzed against a total volume of 1 L of 0.1 M Na -HEPES/HCl pH 7.0 per mL of protein. Final concentration of protein stock solution was fixed at 50 mg/ml as determined by spectrophotometry.

**Figure 4:**
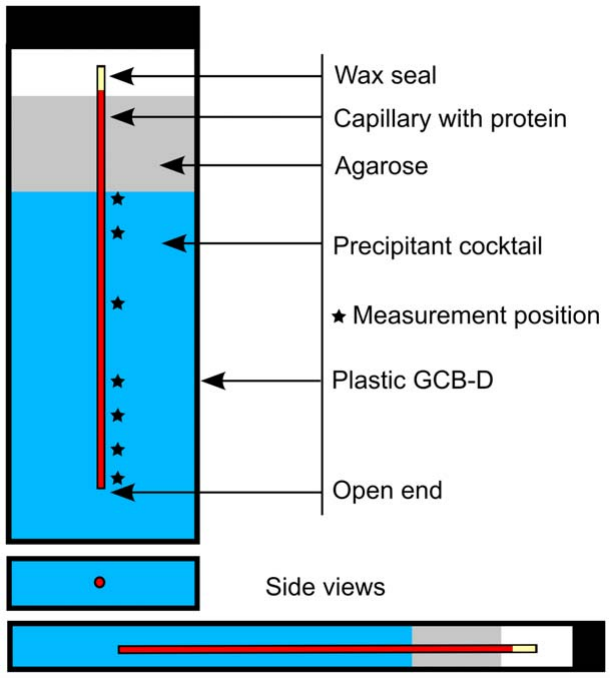
Graphical representation of a typical counter-diffusion experiment set-up in a GCB-Domino (Triana S&T). The dimension of the GCB-Domino is approximately 70 mm high × 17.2 mm wide × 7.0 mm thick. The open end of the capillary allows the precipitant (in blue) to diffuse against the much slower diffusive protein solution, thereby inducing the precipitation of the latter. The capillaries are kept in place by an agarose plug sitting at the top of the precipitant solution. Each mark (*) shows a DLS measurement position, at 0.7, 2.2, 5.1, 9.9, 11.8, 18.7 and 24.5 mm from the open end.

The crystallization condition employed by Carrel and co-workers [44] to crystallize glucose isomerase (solved its structure at 1.9Å) was slightly modified to fulfil the requirements of counter-diffusion experiments [45] by increasing the original concentration of ammonium sulphate from 0.7 M to 3.0 M. Hence, Granada Crystallization Boxes-Domino (GCB-D) reactors [35] containing ammonium sulphate (3.0 M) and Hepes (0.1 M) at pH 7.0 were purchased from Triana S&T and used to set up the crystallization experiments. The GCB-Ds are usually topped with a layer of agarose gel for mechanical stability when the capillaries are punctured (see Figure 4). Then, capillaries of 0.1, 0.2 and 0.3 mm inner diameter were filled with protein stock solution. An extra fourth capillary of 0.3 mm was filled with a mixture of protein solution, buffer and 0.075% *w/v* low melting point agarose (AG-LGT, Triana S&T). All DLS experiments were carried out at RT.

### Set-up of the DLS Instrument

A *Spectro*LIGHT 500 instrument [14] from Nabitec GmbH was used to carry out the DLS measurements. The instrument, which is designed to simultaneously record images and take DLS measurements of crystallization experiments in multiwell plates, had to be adapted to the geometry of the capillaries (e.g. GCB-D, see Figure S1) so that the plane of the incident and reflected beams was perpendicular to the capillary axis. The fluctuation of intensity of the scattered light was recorded by a photomultiplier tube, then the autocorrelation function (ACF) [17] was calculated and interpreted using the CONTIN algorithm [46]. The AFC values obtained using CONTIN were evaluated with the SPECTRO software developed by Nabitec GmbH.

### Procedure for DLS Measurements Inside Capillaries

The crystallization experiment was set up as follows: firstly, each capillary was loaded with protein solution; then one end of the capillaries was sealed with wax and finally the open end was punctured in the gel of the GCB-D. Immediately after, the box containing the capillaries with protein solution was fixed inside the DLS instrument (Figure S1). By means of the instrument’s optical camera it was possible to record the coordinates both at the beginning and at the end of the capillary so that the distance along the capillaries could be converted into the coordinate system of the motorized stage. The DLS signal was recorded in seven different positions of the capillaries over time (see Figure 4 and Movie S1).

At the beginning of the measurements, minute displacements in x, y and z directions were performed at each position to optimize the DLS signal (optimized signal means: the largest count rate at which a smooth ACF with high intercept could be obtained). Subsequently, a series of measurements was set-up (a script was written in Python and given as input to the autopilot of *SPECTRO*), in which a DLS data set was detected for 60 seconds at each of the positions, and then paused for 122 minutes until the beginning of the next cycle. In total, the experiment was monitored during 14 days.

### Solubility and Diffusion Studies for the Simulation Software

The numerical code used for the simulations relies on experimental parameters that relate the solubility of the protein (glucose isomerase) with the concentration of the precipitant (ammonium sulphate). In order to determine the solubility of glucose isomerase, we conducted batch experiments at different concentrations of ammonium sulphate in 100 mM Hepes at pH 7.0 in a volume of 50 µL. After equilibration of the solutions for one month at 20°C, each eppendorf tube was centrifuged and the absorbance of the supernatant was measured at 280 nm. By fitting the absorbance data at each concentration we determined the solubility of Glucose Isomerase using the empirical law 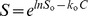 (see Figure S2). From this equation we calculated values of *S_o_* = 173.88 mg/ml and *k_o_* = 3.39 M^−1^ (of AS), which were used to run the simulation software. The diffusion coefficients of glucose isomerase (D_GI_ = 0.53×10^−6^ cm^2^/s [47]) and ammonium sulphate (D_AS_ = 0.9×10^−5^ cm^2^/s [48]) were obtained from literature values.

## Supporting Information

Figure S1 **Photographs of the GCB-D box fixed inside the DLS apparatus.** The orientation of the box was selected so that the perpendicular of the plane of the incident and reflected beams are also perpendicular to the capillary axis.
(TIFF)

Figure S2
**Solubility data of glucose isomerase at 20°C as a function of the concentration of ammonium sulphate.** The data fits the equation of the empirical formula for the solubility 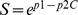, where p1 is the natural logarithm of the solubility at zero ionic strength (S0) and p2 is the salting out constant.(TIFF)

Movie S1 **Progress of a counter-diffusion crystallization experiment by optical microscopy.** The precipitant enters the capillary from the right end. The movie runs at an accelerated time.
(MP4)

Movie S2 **Numerical simulation of Fick’s second law to illustrate the diffusion process contained in the geometry of a capillary.** In the movie, dark red corresponds to a given initial concentration, while dark blue indicates cero concentration.
(MP4)
