# Supplementary figures and images for: Monitoring and Scoring Counter-Diffusion Protein Crystallization Experiments in Capillaries by in situ Dynamic Light Scattering
Source: PLoS One. 2012 Jun 4;7(6):e33545. doi: 10.1371/journal.pone.0033545 (PMC3366972; doi:10.1371/journal.pone.0033545)

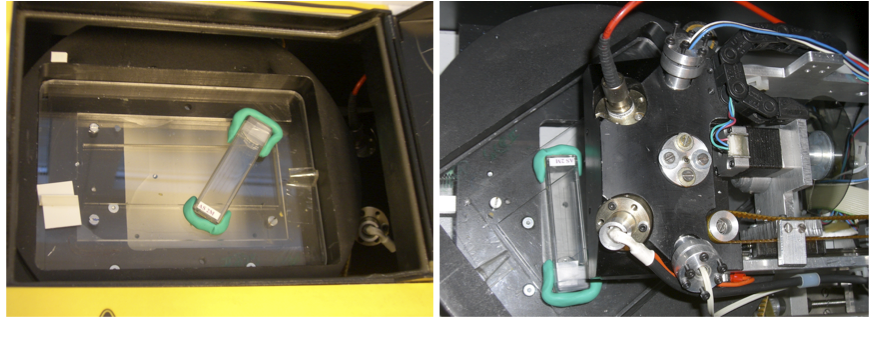

Supplement: Figure S1 — Photographs of the GCB-D box fixed inside the DLS apparatus. The orientation of the box was selected so that the perpendicular of the plane of the incident and reflected beams are also perpendicular to the capillary axis. (TIFF) [file pone.0033545.s001.tif]

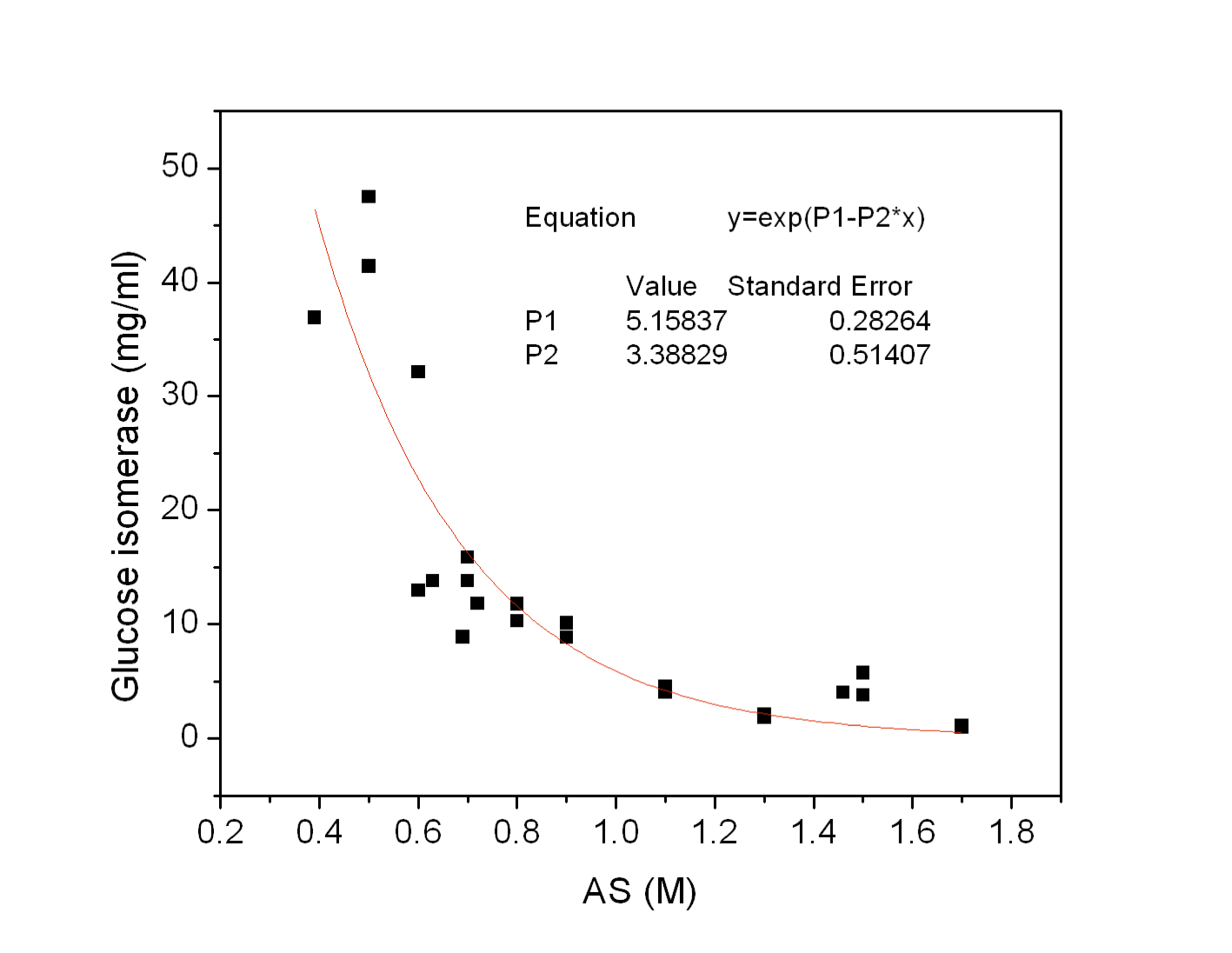

Supplement: Figure S2 — Solubility data of glucose isomerase at 20°C as a function of the concentration of ammonium sulphate. The data fits the equation of the empirical formula for the solubility , where p1 is the natural logarithm of the solubility at zero ionic strength (S0) and p2 is the salting out constant. (TIFF) [file pone.0033545.s002.tif]
